# A Comparison between 2-Octyl Cyanoacrylate and Conventional Suturing for the Closure of Epiblepharon Incision Wounds in Children: A Retrospective Case–Control Study

**DOI:** 10.3390/jcm13123475

**Published:** 2024-06-14

**Authors:** Chia-Chen Hsu, Lung-Chi Lee, Hsu-Chieh Chang, Yi-Hao Chen, Meng-Wei Hsieh, Ke-Hung Chien

**Affiliations:** 1 Department of Ophthalmology, Tri-Service General Hospital, National Defense Medical Center, Taipei 114, Taiwan; water097978kaohsiung9@gmail.com (C.-C.H.); kidday0205@gmail.com (L.-C.L.); doc30879@mail.ndmctsgh.edu.tw (Y.-H.C.); 2 Department of Nursing, Tri-Service General Hospital, National Defense Medical Center, Taipei 114, Taiwan; n3197001@gmail.com; 3 Graduate Institute of Nursing, College of Nursing, Taipei Medical University, Taipei 110, Taiwan; 4 Department of Nursing, Tri-Service General Hospital Beitou Branch, Taipei 112, Taiwan; 5 Department of Ophthalmology, Taoyuan Armed Forces General Hospital, Taoyuan 325, Taiwan

**Keywords:** epiblepharon, 2-octyl cyanoacrylate, tissue adhesive, personal satisfaction

## Abstract

**Background:** Tissue adhesive has been widely used in ophthalmic surgery for various procedures, proving both effective and safe. However, no studies have compared the surgical efficacy of the tissue adhesive 2-octyl cyanoacrylate (SurgiSeal®) to that of traditional suture closure in Asian children undergoing surgery for lower lid epiblepharon. **Methods:** This is a single-center retrospective case-control study. Surgical correction for epiblepharon was performed on 22 patients from November 2019 to May 2023. A total of 20 patients who were followed up for at least 1 month were included for analysis. After standardized epiblepharon surgery, group A underwent wound closure with a subcuticular suture and 2-octyl cyanoacrylate, and group B underwent closure with a 6-O fast-absorbing surgical gut suture. Patients were followed up at 1, 4, and 12 weeks post-surgery. **Results:** A total of 10 patients (20 eyes) underwent skin closure with tissue adhesives (group A), and 10 patients (18 eyes) underwent wound closure using conventional suture material (group B). No significant differences in the sex ratio, mean age at operation, pre- and postoperative best-corrected visual acuity (BCVA), or average surgical time were observed between groups. Both groups exhibited improved postoperative BCVA, with symptom relief and a significant decrease in the severity of keratopathy after surgery. Neither recurrence nor complications were reported during follow-up. The aesthetic results were similar between groups, while caregivers of children in the tissue adhesive group expressed high satisfaction regarding the ease of postoperative care. **Conclusions:** Successful closure of lower lid epiblepharon surgery wounds in children can be performed using 2-octyl cyanoacrylate (SurgiSeal®). This method is simple, safe, and effective when compared to conventional sutures.

## 1. Introduction

Epiblepharon is a congenital lid condition in which the pretarsal orbicularis muscle and eyelid skin override the edge of the eyelid, often leading to eyelash rotation and cornea–eyelash contact. Most of the time, the condition resolves spontaneously when children grow up, but in some cases it may persist and cause corneal damage, astigmatism, blurry vision and other complications requiring surgical correction in addition to conservative management [1,2,3,4,5].

Surgery to correct epiblepharon involves the closure of the eyelid using conventional suture thread, which necessitates careful postoperative care [2,6,7,8]. This care includes daily dressing changes, regular administration of eye drops and ointment, avoiding showering above the face, and refraining from wearing contact lenses. In some cases, suture removal is also required after the surgery.

However, epiblepharon is a congenital or early-childhood-onset condition that typically presents during a stage characterized by heightened activity, difficulties in controlling behavior, and poor adherence to postoperative care. Caregivers often struggle with preventing children from rubbing or touching the incision wound on the eyelid, changing dressings, and administering medications [9,10].

Furthermore, even though most sutures used in epiblepharon surgery are self-dissolving and do not need to be removed, the task of engaging children to cooperate becomes notably challenging when suture removal becomes necessary [11,12].

Wound maintenance in the first few days after surgery depends on the selection of the appropriate surgical thread. In many surgical aspects, suture material is often a prerequisite for successful surgical procedures and proper healing [13,14].

The 2-octyl cyanoacrylate tissue glue has been widely used in ophthalmic surgery, including cataract wound repair, corneal perforation, orbital implant surgery, dysfunctional filtration bleb, and external dacryocystorhinostomy [8,15,16,17,18,19]. It has also been proven to be an alternative to sutures in upper-lid blepharoplasty, with comparable efficacy and complications, as demonstrated in a prospective controlled study [7]. The hypothesis proposed that using 2-octyl cyanoacrylate adhesive for lower eyelid skin wound closure would be as effective as conventional sutures. However, to date, no studies have compared the efficacy, safety, and aesthetic outcomes of 2-octyl cyanoacrylate tissue glue with conventional suturing for the closure of surgical wounds in epiblepharon correction.

## 2. Materials and Methods

This retrospective case–control study involved reviewing the medical records of patients with epiblepharon aged younger than 18 years who underwent surgical correction at Tri-Service General Hospital in Taipei, Taiwan, between 1 November 2019 and 31 May 2023. Institutional board review (C202305032) approval to review patient records and the need for informed consent was waived. 

This study was conducted in accordance with the Declaration of Helsinki and the STROBE guidelines (see Supplementary Table S1).The procedures for correcting epiblepharon over the lower lids were identical except for the modality employed to secure the closure of the skin. A total of 12 and 10 patients underwent eyelid closure with 2-octyl cyanoacrylate adhesive (SurgiSeal^®^; Adhezion Biomedical, LLC., Wyomissing, PA, USA) (group A) and 6-O catgut suture (Ethicon; Johnson and Johnson, Livingston, UK) (group B), respectively. Surgery was performed by one operator (K.-H. Chien) after obtaining consent for surgery. The indication for surgery is symptomatic patients with a poor response to conservative treatment, such as topical lubricant or corneal damage due to eyelash–cornea touch and decreased visual acuity. Patients without at least 1 month of postoperative follow-up were excluded. The data reviewed encompassed various factors, such as age at the time of the operation, gender, symptoms, surgical time, the affected side of epiblepharon, associated ocular diseases, refraction, visual acuity, and ocular surface assessment (including external eye photo and fluorescein eye stain test) before and after the surgery. Additionally, complications, including wound dehiscence, infection, scar formation, lid ectropion or retraction, as well as the rate of recurrence, were evaluated. A successful outcome was defined as the resolution of the presenting indication for epiblepharon correction and in the absence of recurrence.

### 2.1. Technique

**Figure 1:**
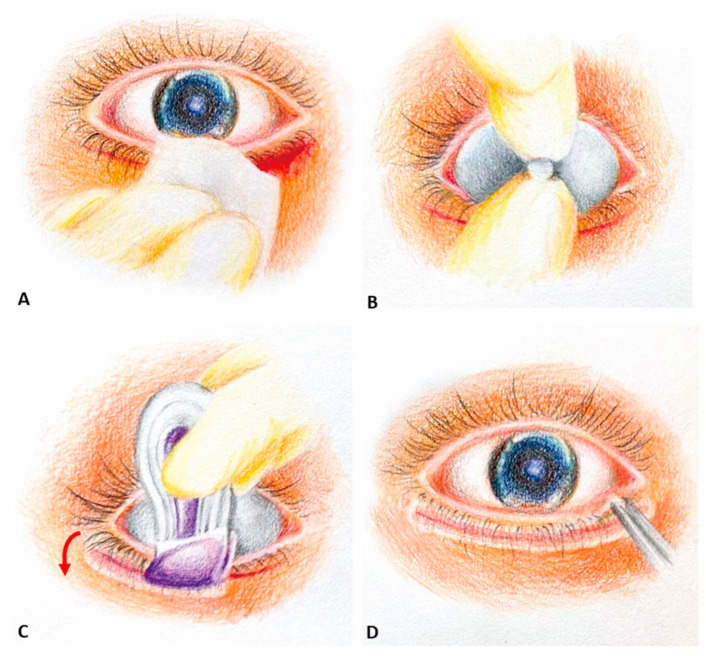
Surgical procedure illustration. (**A**) After performing the subcuticular suture using a 6-O fast-absorbing surgical gut suture, any blood stains present around the wound area were cleared using wet gauze. (**B**) To safeguard the ocular surface, a sterilized corneal shield was inserted. (**C**) The SurgiSeal^®^ applicator’s sponge was oriented downward, and its perforated line aligned vertically with the incision wound (the red arrow indicates the direction of application). Two to three thin layers of 2-octyl cyanoacrylate were applied, allowing 15 s for each layer to dry. The eyelashes were simultaneously brushed downward during this process. (**D**) Meticulously check and remove residual tissue glue in undesired locations.

Epiblepharon correction followed the procedure described by Woo et al. [20] under general anesthesia. The process began with marking the incision area and injecting 1% lidocaine with 1:200,000 adrenaline (Lidophrine injection; Oriental Chemical Works INC. Taipei, Taiwan) for hemostasis. A subciliary incision was made approximately 1–2 mm below the eyelashes across the entire eyelid. Excess skin and a small part of the orbicularis muscle were removed, and bipolar electrocautery ensured hemostasis. The skin–orbicularis oculi muscle flap was separated from the tarsal plate, and three to six 6–0 Vicryl sutures (Ethicon; Johnson and Johnson, Livingston, UK) were used to secure the tarsal plate to the subcutaneous tissue, focusing more on the medial site where the epiblepharon was prominent, with the aim of altering the direction of the eyelashes. For group A, continuous subcuticular 6-O fast-absorbing surgical gut sutures were used to approximate skin edges. After cleaning and drying the surgical site, a sterilized corneal shield (autoclavable corneal shield pair with handle; Kolberg Ocular Supplies, INC. Bella Vista, AR, USA) was inserted, and the skin edges were sealed with 2-octyl cyanoacrylate. With the sponge of the SurgiSeal^®^ applicator facing downward and the perforated line vertical to the incision wound, two to three thin liquid layers of 2-octyl cyanoacrylate were applied with 15 s of dry time in between. Simultaneously, brushing the lashes downward during the application process can aid in averting contact between the eyelashes and the cornea, thereby reducing the risk of such contact-induced corneal damage. Unintentional application over the ocular surface should be avoided during the process, and any residual tissue glue in undesired locations, such as the cornea, bulbar or palpebral conjunctiva, should be meticulously checked and removed. (Figure 1) For group B, primary closure with 6-O catgut suture was performed after the everting buried 6–0 Vicryl sutures were placed.

### 2.2. Postoperative Care Instructions

For eyelid closure with 2-octyl cyanoacrylate, a combination of eye drops containing a corticosteroid and an antibiotic (Tobradex^®^ by Alcon Laboratories, Ft. Worth, TX, USA) was administered four times per day. Covering the wound with ointment or mineral oil was avoided. It is fine to shower or wash the face, including the eyelid. The tissue glue covering the wound should be allowed to shed spontaneously; it should not be removed by rubbing or tearing.

For eyelid closure with absorbing gut sutures, eye drops with Tobradex^®^ were applied four times per day, along with tetracycline ointment (Tetracycline Hydrochloride Ophthalmic Ointment by Genuine Chemical Pharmaceutical Co., Ltd. Taoyuan County, Taiwan) twice daily, to the incision wound for 2 weeks. The incision site should be kept clean and dry. Cleaning the suture lines with sterile 0.9% normal saline solution once a day, showering below the neck, washing the face with wipes below the eyes and avoiding rubbing the wound were recommended for 1 week following surgery. Patients were followed up in the outpatient clinic at 1, 2, and 4 weeks post-operation.

### 2.3. Patient-Reported Outcomes

At 1 month post-surgery, an online questionnaire was administered to evaluate the satisfaction of patients or their parents. A visual analog scale was utilized to quantitatively assess satisfaction with esthetic outcomes, symptom relief, the ease of postoperative care, and general satisfaction. The scale ranged from 0 (completely dissatisfied) to 10 (highly satisfied). This scale has been demonstrated to be an effective tool for quantifying subjective parameters and facilitating statistical analysis [21,22].

### 2.4. Statistical Analysis

Demographic data were analyzed using descriptive statistics. Continuous variables are presented as the mean ± standard deviation. The Mann–Whitney U test was used to compare the continuous variables between groups. To assess intragroup pre- and post-visual acuity changes, the Wilcoxon signed rank test was utilized. SPSS for Windows (version 18.0; SPSS Inc., Chicago, IL, USA) was used.

## 3. Results

A total of 22 patients underwent epiblepharon correction surgery during the study period, but two patients using 2-octyl cyanoacrylate were excluded due to failure to follow up after surgery.

Group A included 20 eyes from 10 patients, and group B comprised 18 eyes from 10 patients. All patients were Taiwanese and had no previous operations for epiblepharon. Both groups had an equal distribution of boys and girls (5:5). The mean age in group A was 7.9 ± 2.2 years (range: 5–12 years), while in group B it was 7.2 ± 4.2 years (range: 3–16 years). There was no significant difference in the sex ratio, mean age at the operation, or mean body mass index between the two groups.

All patients in group A and 80% of the patients in group B underwent surgery on bilateral lower lids. The average duration of surgeries for groups A and B were 27.6 ± 7.7 and 30.9 ± 10.0 min per eye, respectively. However, no significant difference between the two groups was observed (*p* = 0.334). Out of the patients in group A, 50.0% exhibited astigmatism exceeding 1.0 diopter, while in group B, this proportion was higher at 83.3%. The mean baseline best-corrected visual acuity (logMAR) of groups A and B was 0.19 ± 0.21 and 0.18 ± 0.07, respectively (*p* = 0.568), and the mean postoperative best-corrected visual acuity of groups A and B was 0.05 ± 0 and 0.11 ± 0.03, respectively (*p* = 0.442). Notably, postoperative best-corrected visual acuity showed improvement in both groups compared to the preoperative values. The mean follow-up time for group B was significantly longer (5.8 ± 6.9 months) than that for group A (1.5 ± 0.9 months) due to more children in group B having associated ocular disorders such as amblyopia, strabismus, and high astigmatism, which necessitated long-term follow-up and management (Table 1).

**Figure 2:**
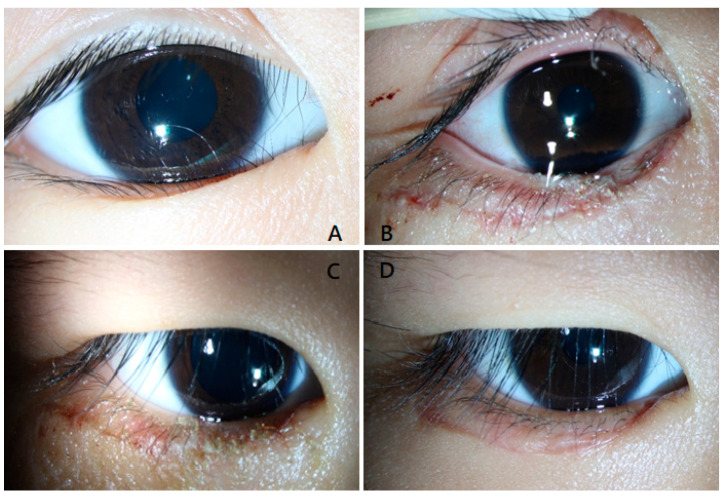
External eye photography of a patient in group A. ((**A**) preoperative; (**B**) postoperative day 1; (**C**) postoperative day 7; (**D**) postoperative day 30).

All patients in both groups experienced symptom resolution after the operation. The aesthetic outcome and inflammatory level were similar between the groups, but crust formation in group A was significantly less than that in group B. The cilia of the SurgiSeal-treated eye were initially glued to the skin in a downward direction, and we observed that the lashes remained in the same direction even after the glue fell off after 10–14 days (Figure 2). In contrast, some cilia in the sutured group may lift away from the skin and change direction after the stitches are absorbed.

Following a minimum of 1 month of follow-up, all cases of epiblepharon were well corrected. Neither group reported any cases of recurrence or complications, including wound dehiscence, suture infection, scar formation, lid retraction, or lid ectropion.

**Figure 3:**
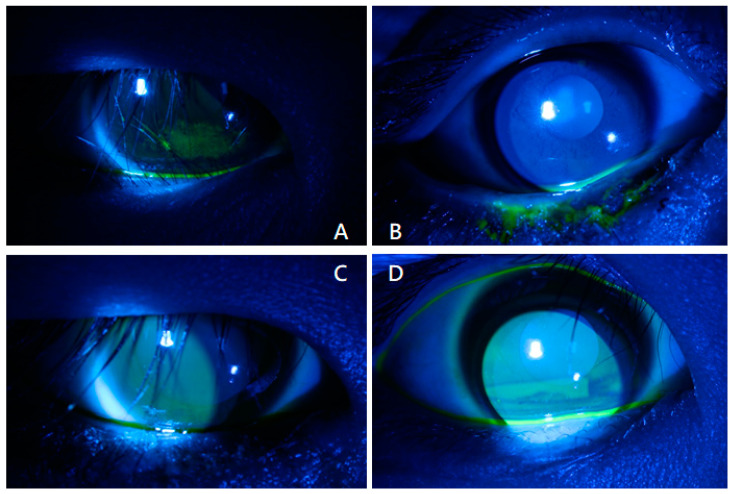
Phots of fluorescein eye stain test of a patient in group A. (**A**) Pre-operatively, an apparent corneal abrasion was observed over the inferior third of cornea due to cornea–eyelash contact. (**B**) On postoperative day 1, epiblepharon was corrected, and the corneal surface had recovered without any damage. (**C**) Postoperative day 7 and (**D**) postoperative day 30 showed no signs of corneal abrasion.

A fluorescein eye stain test was performed during every follow-up visit. The corneal damage resulting from epiblepharon in most cases improved rapidly post-operation. Only one case had delayed healing of corneal abrasion and resolution of corneal haze despite a well-corrected direction of eyelash due to suspected limbal insufficiency (Figure 3).

Postoperative satisfaction results are presented in Table 2. Both patients and caregivers reported satisfaction with the final cosmetic outcomes and symptom relief across both groups. However, the satisfaction regarding the ease of postoperative care and overall satisfaction were significantly higher in Group A compared to Group B (*p* < 0.05). Additionally, while no formal questionnaire was administered, satisfaction levels were assessed through interviews during each follow-up visit. The majority of caregivers within group A expressed a high level of satisfaction with the simplicity of postoperative wound care. This contentment stems from the water-resistant and nonirritating properties of the tissue glue, which eliminated the requirement for dressings or application of ointments to the wound.

Notably, one patient visited this department with complaints of rapid progression of myopia even under 0.1% atropine and expressed a desire for orthokeratology. However, upon observing a significant area of corneal abrasion associated with epiblepharon, we recommended surgical correction as the initial approach. Orthokeratology was subsequently employed 2 weeks after the surgery, as corneal damage had resolved, and myopic progression was under control afterward. Preoperative high astigmatism (>3 diopters) noted in another two cases in group A decreased substantially 1 month post-operation.

## 4. Discussion

2-Octyl cyanoacrylate, a type of synthetic adhesive, has gained significant popularity since its approval by the U.S. Food and Drug Administration in 1998 [23]. It has emerged as a promising alternative to sutures due to its numerous advantages. This adhesive is nontoxic, biocompatible, flexible, and possesses antimicrobial properties and high tissue bonding strength [24,25]. Moreover, it induces low inflammatory and foreign body responses, making it an excellent choice for medical applications. By using this adhesive, several shortcomings associated with sutures can be avoided, including wound dehiscence, inflammation, microbial infection, irritation, and vascularization.

Currently, 2-octyl cyanoacrylate is widely used as a wound sealant in children, particularly in emergency departments [26,27,28]. Its quick application process provides comfort to patients while ensuring acceptable cosmetic outcomes. Additionally, it eliminates the need for sutures and subsequent suture removal, thereby streamlining the healing process [11].

This current study confirms the effectiveness of 2-octyl cyanoacrylate (SurgiSeal) in lower lid epiblepharon surgery. Both groups required the same operative time and demonstrated successful correction of epiblepharon with equally desirable cosmetic outcomes and comparable complication rates. 2-Octyl cyanoacrylate (SurgiSeal) is easy to apply and offers a flexible and water-resistant protective coating with antimicrobial properties. Additionally, it eliminates the need for dressings and suture removal, leading to high levels of postoperative satisfaction. It also enables early access to orthokeratoplasty or myopic control contact lenses for children with rapid myopia progression.

Regarding its application in ophthalmology, Taravella et al. [29] reported a successful case of treating corneal perforation with 2-octyl cyanoacrylate in 2001. Since then, this adhesive has been utilized in various ocular conditions, such as glaucoma leakage filtration bleb, sealing cornea cataract wounds, retinal detachment surgery, and orbital surgery, and in most studies, Dermabond^®^ (Ethicon) was used [30,31,32].

In the context of eyelid surgery, Grrence et al. [7] conducted a prospective controlled study comparing the closure of upper-lid blepharoplasty using 2-octyl cyanoacrylate (Dermabond) with conventional sutures. The study revealed that both methods yielded equivalent aesthetic outcomes and showed no significant differences in complications or inflammation levels. However, it is worth noting that using Dermabond might require specific techniques and comes with some limitations. During the application of 2-octyl cyanoacrylate, an assistant holds Castroviejo forceps to approximate the skin borders, and in some cases sutures are necessary to facilitate the application process. However, they suggested that using Castroviejo forceps for this purpose takes more time than suturing.

Moreover, in the application of Dermabond, a siliconized tuberculin syringe was found to be a much easier, faster, and more precise tool than the Dermabond applicator itself. Additionally, in three cases, using the Dermabond applicator resulted in glue leaching into the wound. Another study also reported that excess glue remained around the wound when using the Dermabond applicator [33]. These findings suggest that while the Dermabond applicator is usable, it may not be the most ideal tool for eyelid wound closure.

In this study, we utilized SurgiSeal as a surgical sealant. SurgiSeal demonstrates similar adhesive strength and flexibility when compared to Dermabond. Furthermore, it exhibits superior peel strength and wound closure strength in contrast to Dermabond [34,35,36]. Additionally, SurgiSeal offers superior moisture permeability, promoting improved healing properties over Dermabond [36].

We found that SurgiSeal is particularly suitable for application to lower lid wounds for several reasons. When performing upper-lid blepharoplasty, the eyelashes are situated far from the eyelid crease, making the application of tissue glue easier. However, for the lower lid, the subciliary incision line is near the cilia, necessitating cautious application. To address this, we adapted our technique to achieve optimal results.

First, SurgiSeal’s large application area allows for efficient coverage, with the perforated line diameter matching approximately half of the length of the lower lid. We applied SurgiSeal along the perforated line, running vertical to the wound, from the eyelid margin to the lower border of the wound. Second, each brush stroke was used to gently comb the eyelashes away from the cornea, ensuring that they lie flat and are securely fixed on the skin. Last, the controlled slow release of liquid SurgiSeal from the applicator bubble to the sponge prevented any adhesive material from spilling or running sideways onto the surrounding tissue.

In previous studies using 2-octyl cyanoacrylate for wound closure in upper-lid blepharoplasty [7,33], no subcuticular sutures were used. However, in some cases, interrupted or temporary sutures were necessary to approximate the skin edges before applying the tissue adhesive. On the other hand, in this study, for the closure of the lower lid wound, although superficial and has low tension, we employed tissue adhesive in conjunction with subcuticular sutures. This decision was made because the surgical site had a small area, and attempting to approximate the skin edges with fingers or forceps could result in excessive tension, leading to an uneven surface of the wound. This, in turn, could potentially cause ectropion after the tissue adhesive dries. Moreover, the proximity of the surgical incision to the cilia of the lower lid made it challenging to hold the wound edge with forceps or other instruments without disturbing the direction of the eyelashes. Any disturbance could raise the eyelashes, potentially damaging the cornea and causing discomfort to the patient.

Additionally, the subcuticular suture also played a crucial role in preventing the infiltration of tissue adhesive into the wound. Such infiltration could delay the healing process and negatively impact the overall outcome of the procedure [7]. At the same time, the operative time did not show a significant difference between the two groups, indicating that the subcuticular suture did not delay the operative time.

To minimize the risk of iatrogenic damage to the anterior segment and to enhance patient comfort, a cornea shield was carefully inserted before applying the tissue glue. This precautionary measure was taken because the eyelid margin is near the skin incision of the lower lid. By using the cornea shield, the delicate cornea was protected, and any potential adverse effects from the tissue glue were reduced. Additionally, the use of the cornea shield eliminated the necessity for patients to wear a bandage contact lens.

The majority of patients undergoing surgery were between the ages of 5 and 16 years old, which aligns with findings from previous studies [3,20]. The positive effects of blepharoplasty on visual acuity improvement and reduced astigmatism are also consistent with previous research results [4,37,38].

Notably, no complications were observed in either group during the present study, and minimal inflammation was noted post-operation. In a study conducted by Ahn et al. [39] using rabbits, it was found that 2-octyl cyanoacrylate adhesive showed similar efficacy in closing upper eyelid incision wounds with comparable macroscopic outcomes. Moreover, the use of tissue adhesive resulted in less inflammation reaction and similar fibrosis degree in histopathology at 2 months after incision compared to 7-0 nylon sutures.

In our study, fast-absorbing catgut sutures were utilized in both groups, and we hypothesized that the inflammatory degree would be similar between the groups and potentially higher than that with non-absorbing nylon sutures. Further investigation is warranted to explore this aspect in more detail.

While tissue adhesive is generally considered safe, complications can still arise. Previous reports have associated cyanoacrylate tissue adhesive with conditions such as symblepharon, infection, granulomatous keratitis, or giant papillary conjunctivitis, mostly occurring when the adhesive comes into direct contact with the cornea and conjunctival tissues [40,41,42].

In our study, however, no complications related to cyanoacrylate tissue adhesive were observed. This can be attributed to our meticulous approach in applying the tissue glue, using a corneal shield to protect the delicate cornea, and promptly identifying and removing any residue glue that was inadvertently placed in unwanted areas.

Both groups displayed similar subjective satisfaction regarding cosmetic outcomes and symptom relief. Furthermore, patients who received 2-octyl cyanoacrylate adhesive reported significantly higher satisfaction with the ease of postoperative care and overall satisfaction compared to those who received traditional sutures. These findings are consistent with previous studies [28,43]. Tsai et al. [43] reported that general satisfaction was higher with SurgiSeal compared to conventional sutures for the treatment of facial lacerations in children. Thus, 2-octyl cyanoacrylate presents a valuable alternative to conventional sutures.

This study has several limitations that should be considered. First, the sample size was small, and this study only included participants from a single country. Therefore, additional clinical trials with larger and more diverse populations may be warranted. Additionally, this was a retrospective review of medical records. A prospective study with age-matched participants or a randomized controlled trial, where each patient serves as their own control, is needed to eliminate confounding factors such as skin variation among different participants. Nevertheless, the similarity in fundamental characteristics among the enrolled participants, as illustrated in Table 1, mitigates selection bias. Additionally, the findings align with published data on cyanoacrylates, demonstrating an excellent safety profile and effective outcomes. Second, the long-term outcomes were not assessed, and it has been reported that epiblepharon could recur 1–2 years post-surgery, which warrants further investigation [20]. Another limitation is that SurgiSeal is more expensive than sutures (each vial of SurgiSeal tissue adhesive costs USD 185.8, compared with USD 4.95 for a 6-O catgut suture in this hospital). However, the higher cost may be justified by the ease of postoperative care and the elimination of the need for wound dressing as well as suture removal. The cost-effectiveness of cyanoacrylate in facial lacerations in children has also been reported in a previous study [44,45,46].

## 5. Conclusions

In conclusion, 2-octyl cyanoacrylate is a surgical sealant that represents a safe approach for wound closure in the surgical correction of epiblepharon among pediatric patients. It provides outcomes comparable to the traditional suture method while offering a host of additional benefits. These advantages include the resolution of presenting symptoms, improvements in visual acuity, and high levels of satisfaction among both patients and caregivers.

## Figures and Tables

**Table 1:** Patient demographic characteristics

Characteristic	Group A	Group B	*p* Value
Gender			
Male	5	5	
Female	5	5	
Mean age at operation (years)	7.9 ± 2.2	7.2 ± 4.2	0.303
Average body mass index (kg/m^2^)	18.97 ± 4.18	17.55 ± 4.85	0.335
Side of epiblepharon			
Unilateral	0	2	
Bilateral	10	8	
Mean operation time (per eye) (minutes)	27.6 ± 7.7	30.9 ± 10.0	0.334
Associated ocular disease (eyes)			
Amblyopia	2 (3)	3 (6)	
Strabismus	0	2 (4)	
Congenital ptosis	0	1 (1)	
Astigmatism (>1 diopter)	6 (10)	8 (15)	
High astigmatism (>3 diopter)	1 (2)	2 (4)	
Myopia	4 (8)	4 (8)	
Preoperative BCVA (logMAR)	0.19 ± 0.21	0.18 ± 0.07	0.568
Postoperative BCVA (logMAR)	0.05 ± 0	0.11 ± 0.03	0.442
Mean follow-up time (months)	1.5 ± 0.9	5.8 ± 6.9	0.463

BCVA—best-corrected visual acuity.

**Table 2:** Treatment satisfaction.

	Group A	Group B	Z *	*p* Value
Esthetic outcomes	9 ± 0.82	8.9 ± 0.74	−0.284	0.776
Symptom relief	9.6 ± 0.52	9.5 ± 0.53	−0.438	0.661
Ease of postoperative care	9.1 ± 0.74	6.9 ± 0.99	−3.629	<0.001
General satisfaction	9.1 ± 0.32	8.1 ± 0.57	−3.482	<0.001

Note: * Mann–Whitney U test.

## Data Availability

Data are available from the corresponding author on reasonable request.

## Supplementary Materials

The following supporting information can be downloaded at: https://www.mdpi.com/article/10.3390/jcm13123475/s1, Table S1: STROBE checklist of items of case-control studies.
